# Editorial: An overview on allergic and pulmonary diseases: from birth to childhood

**DOI:** 10.3389/fmed.2023.1353772

**Published:** 2024-01-10

**Authors:** Sara Manti, Antonella Gambadauro, Alessandra Li Pomi, Salvatore Leonardi

**Affiliations:** ^1^Pediatric Unit, Department of Human Pathology of Adult and Childhood Gaetano Barresi, University of Messina, Messina, Italy; ^2^Pediatric Respiratory Unit, Department of Clinical and Experimental Medicine, University of Catania, Catania, Italy

**Keywords:** allergy, diagnosis, pulmonology, pulmonary diseases, childhood

Allergic and pulmonary diseases are common chronic diseases in the general population including children. They can occur from very early childhood or later; then, they can remain stable or evolve into other forms until adulthood. Their prevalence and economic burden are increasing worldwide despite therapeutic advances. Thus, the management of allergic and pulmonary diseases has become an essential issue in the medical field. In parallel, pediatricians have had to change their approach to many allergic and pulmonary diseases since the latter requires constant updating, both from a pathogenic and therapeutic perspective. Pediatric allergy and pulmonology are highly relevant since many overlapping areas require a combined approach that leads to a complete understanding of the disease processes.

This Research Topic aimed to update knowledge on pathogenesis, clinical presentation, diagnosis, and treatment of allergic and pulmonary diseases, especially related to the pediatric population. Finally, in the era of the COVID-19 epidemic, any research on the diagnostic and therapeutic management of patients suffering from allergic and or pulmonary diseases who have encountered the SARS-CoV-2 is especially welcome. Therefore, this Research Topic also aimed to collect and share early clinical experiences on this new infectious disease and create awareness for early recognition of this disease, especially in critically ill patients.

In their narrative review, the authors highlighted the importance of understanding the pathophysiology of respiratory diseases, such as asthma and allergic rhinitis (AR), as it is crucial in developing novel therapies to treat this incurable disease, which is often comorbid with other airway diseases (Klain et al.; Nur Husna et al.). Accordingly, Malizia et al. hypothesized that a cluster analysis based on the evaluation of cytokines in nasal lavage (NL) could characterize distinctive Seasonal Allergic Rhinitis (SAR) endotypes in a pediatric population. Accordingly, children were grouped in clusters by using interleukin (IL)-5, IL-17, IL-23, and Interferon (INF)-γ in NL, and, three SAR endotypes were identified as follows: cluster 1 showed lower levels of IL-5 and IL-17 and intermediate levels of IL-23 and IFN-γ; cluster 2 had higher levels of IL-5 and intermediate levels of IL-17, IL-23, and IFN-γ; and cluster 3 showed higher levels of IL-17, IL-23, and IFN-γ and intermediate levels of IL-5. The findings suggest that characterizing specific endotypes may offer a more precise description of inflammatory patterns than phenotypes alone. This distinction could have potential clinical implications, especially concerning the development of personalized therapeutic approaches.

Due to advancements in understanding the pathophysiology of respiratory diseases, the diagnostic and therapeutic approaches to pediatric respiratory conditions have significantly changed in the last two decades. This progress has also led physicians and researchers to confront new challenges in pediatric respiratory diseases. The pathophysiological role of childhood diseases throughout the lifespan has seen several changes, highlighting that respiratory diseases in adulthood are significantly affected by events occurring during prenatal and early life, as well as environmental exposures. Therefore, the disorders previously categorized as “adult pathologies” or “rare,” such as cystic fibrosis and chronic obstructive pulmonary disease (COPD), are also the result of long-term consequences of premature birth, congenital and or genetic diseases of the respiratory tract (Steinke et al.; Zhang et al.; Nolasco et al.).

Ensuring the best possible education for emerging pediatric pulmonologists is crucial to guiding them in caring for sick children and the evolving landscape of respiratory diseases throughout adulthood. A “global” and “integrated” point of view is crucial for effectively addressing the healthcare needs of present and future populations.

## Author contributions

SM: Supervision, Visualization, Writing—original draft, Writing—review & editing. AG: Visualization, Writing—original draft. AL: Visualization, Writing—original draft. SL: Resources, Supervision, Validation, Visualization, Writing—review & editing.

